# Improved Sampling of Adaptive Path Collective Variables by Stabilized Extended-System Dynamics

**DOI:** 10.1021/acs.jctc.3c00938

**Published:** 2023-12-11

**Authors:** Andreas Hulm, Christian Ochsenfeld

**Affiliations:** † Chair of Theoretical Chemistry, Department of Chemistry, LMU Munich, Butenandtstr. 5, München D-81377, Germany; ‡ Max Planck Institute for Solid State Research, Heisenbergstr. 1, Stuttgart D-70569, Germany

## Abstract

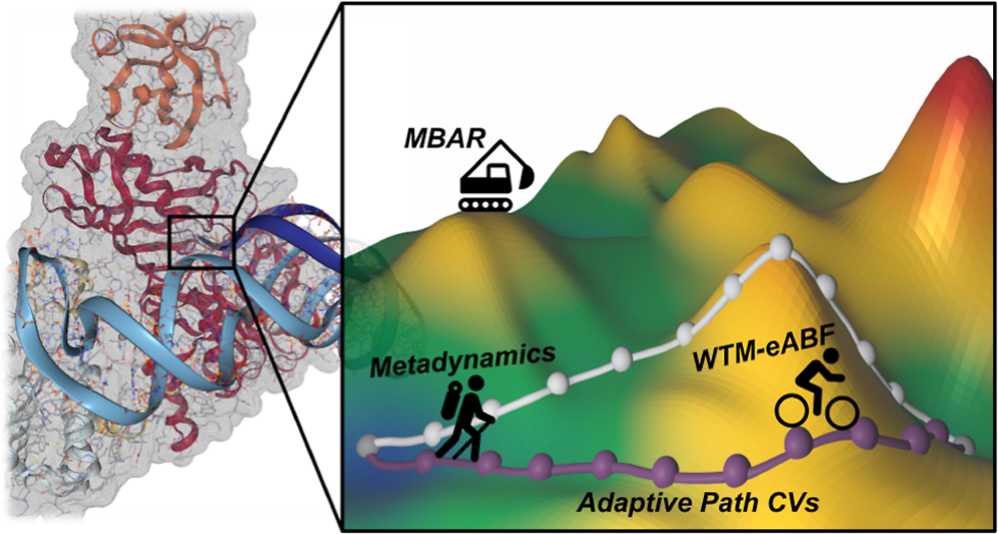

Because of the complicated multistep nature of many biocatalytic reactions, an a priori definition of reaction coordinates is difficult. Therefore, we apply enhanced sampling algorithms along with adaptive path collective variables (PCVs), which converge to the minimum free energy path (MFEP) during the simulation. We show how PCVs can be combined with the highly efficient well-tempered metadynamics extended-system adaptive biasing force (WTM-eABF) hybrid sampling algorithm, offering dramatically increased sampling efficiency due to its fast adaptation to path updates. For this purpose, we address discontinuities of PCVs that can arise due to path shortcutting or path updates with a novel stabilization algorithm for extended-system methods. In addition, we show how the convergence of simulations can be further accelerated by utilizing the multistate Bennett’s acceptance ratio (MBAR) estimator. These methods are applied to the first step of the enzymatic reaction mechanism of pseudouridine synthases, where the ability of path WTM-eABF to efficiently explore intricate molecular transitions is demonstrated.

## Introduction

The computation of reliable reaction and activation-free energies of biocatalytic reactions requires extensive sampling of molecular transitions,^1,2^ which is typically obtained using ab initio molecular dynamics (MD) simulations on composite quantum mechanics/molecular mechanics (QM/MM) level of theory.^3^ Using these highly costly simulations, only time scales of up to several 100 ps can be reached, which means that reactive events that are separated by high free energy barriers can never be observed in conventional MD trajectories. It is therefore paramount to apply importance-sampling strategies that speed up the exploration of high-energy regions.^4^

Many of these methods rely on the definition of a low-dimensional set of collective variables (CVs) that discriminate between reactant and product states.^5−8^ For example, metadynamics (MtD) accelerates the exploration of predefined CVs with an adaptive bias potential that builds up during the simulation.^7,9^ However, a bad choice of CV results in hysteresis and poor convergence of the free energy estimate.^10^ For simple transitions, it can be straightforward to find sufficient CVs based on chemical intuition, but for complicated enzymatic processes, the definition of good CVs that contain all the slow degrees of freedom of the given process can become exceedingly difficult. Additionally, CV space is typically limited to up to 3 dimensions because of the exponential growth of computational cost with the number of dimensions,^11^ which is insufficient for complicated multistep transitions.

This problem motivates large research efforts to design one-dimensional CVs that can describe transitions with many slow degrees of freedom, for example, utilizing machine learning methods^12−15^ or path CVs (PCVs).^16−19^ For the latter, a path is defined by a string of discrete nodes that connect metastable states, and the CV is given by a progress parameter. This not only allows for a smooth, one-dimensional parametrization of complex transitions but also provides the opportunity for systematic on-the-fly improvement by iteratively moving a guess path closer to the minimum free energy path (MFEP).

In this contribution, we will build on path MtD as formulated by Ensing and co-workers.^18,19^ We show how MtD can be replaced with the more efficient well-tempered metadynamics extended-system adaptive biasing force (WTM-eABF)^20,21^ hybrid algorithm. Additionally, we show how postprocessing of path WTM-eABF with the multistate Bennett’s acceptance ratio (MBAR)^22^ estimator, which recovers the unbiased statistical weight of each simulation frame, can further improve the convergence of adaptive path simulations. To this end, after a short theoretical overview of the path WTM-eABF algorithm, we compare its performance to that of conventional path MtD on a numerical Müller–Brown (MB) potential. Afterward, we showcase the application of path WTM-eABF on the biocatalytic reaction mechanism of pseudouridine synthases (PUS), which involves a challenging rotating motion of an unbound uridine.

## Theory

Let ξ(**x**) be some CV that represents the reaction coordinate and connects two metastable states. For a finite value *z* of the CV, it has the marginal probability distribution
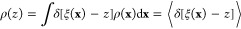
1where ⟨⟩ denotes the ensemble average and δ[*x*] denotes the Dirac delta distribution.^1^ Our goal is to efficiently estimate ρ(*z*), which defines the potential of mean force (PMF) (i.e., free energy profile), according to
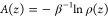
2where  and *k*_B_ is the Boltzmann constant.

For this purpose, MtD builds a repulsive potential
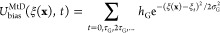
3by adding Gaussian hills with height *h*_G_ and variance σ_G_ in regular time intervals τ_G_, pushing the system away from already explored regions of CV space.^23^ To ensure smooth convergence of *U*_bias_^MtD^, WTM adds an exponential scaling factor  with an effective bias temperature Δ*T* to decrease the height of new Gaussians over time.^9^ Upon convergence, the PMF can be directly obtained from the inverse of the bias potentials.

In contrast, in the closely related WTM-eABF sampling algorithm,^20,21^ a MD simulation of the extended system (**x**, λ) is performed in the potential
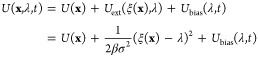
4where the molecular potential energy function *U*(**x**) is coupled to an extended variable λ by a harmonic potential with thermal coupling width σ. Typically, small values of σ are chosen to ensure a tight coupling of λ to ξ(**x**). This framework offers high flexibility and robustness against the choice of bias potential, *U*_bias_(λ, *t*), which only acts on λ and has no direct impact on the physical system. In WTM-eABF, a combination of the WTM bias potential and an adaptive biasing force (ABF^8,24^), which is obtained as the average force acting on λ at a certain value of the CV, is applied to ensure fast exploration of the reaction coordinate.^21^

In practice, the biggest shortcoming of both MtD/WTM and WTM-eABF is their dependence on the choice of CVs, which are usually defined a priori. Bad choices of CVs lead to significant artifacts of sampling and free energy estimates.^10^ In addition, it was shown that activation free energies and reaction rates are particularly vulnerable,^25^ which are among the most important targets of mechanistic studies. One promising direction to mitigate this problem is the development of systematically improvable CVs like adaptive PCVs, which exist in various different flavors.^17−19,26^ Here, we apply geometric PCVs as proposed by Ensing and co-workers,^18,19^ although the presented framework is also valid for other formulations. A path is defined by *M* discrete, equidistant nodes that are placed along the reaction coordinate. The progress *s*(**z**) along the path in the space of some selected CV space **z** = (ξ_1_(**x**), ξ_2_(**x**), ...) can be defined by
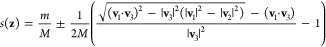
5where *m* is the zero-based index of the closest node and vectors **v**_1_, **v**_2_, and **v**_3_ are defined by **v**_1_ = **z**_*m*_ – **z**, **v**_2_ = **z** – **z**_*m*–1_, and **v**_3_ = **z**_*m*+1_ – **z**_*m*_. The ± in eq 5 is positive if *z* is left of the closest path node and a negative sign otherwise.

Thus, in eq 4, λ is now coupled to the progress parameter *s*(**z**) instead of a simple CV. We note that this definition does not completely eliminate the problem of manually selecting relevant CVs but rather replaces it with the more flexible choice of an appropriate CV space. To avoid the definition of a CV space, one could alternatively apply the arithmetic PCV as formulated by Branduardi and co-workers.^17^ More details on our PCV implementation are given in Section S1 of the Supporting Information.

To converge the path to the MFEP, initial guess nodes are adapted to the average CV density perpendicular to the path by updating the node positions according to
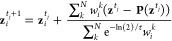
6where  denotes node positions after the last update and the weight of the update for node *i* in step *k* is given by
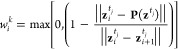
7which is only nonzero for the two closest nodes.^19^ The half-life of the weight of the original path can be chosen with the parameter τ. **P**(**z**) denotes the projection of **z** on the path. In practice, weights *w*_*i*_^*k*^ and the average distance from the path are accumulated between updates, which are applied for every *N*-th step. The initial path might be obtained, for example, by linear interpolation between end points or with some zero-temperature path optimization method (e.g., nudged elastic band method^27^). The latter might additionally already provide some mechanistic information that supports the user in the manual selection of a suitable CV space. After every update, the path is reparametrized to ensure equidistant spacing of nodes, as described in the Supporting Information. To judge the convergence of the PCV, we monitor the quantity
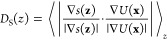
8which approaches exactly zero at the transition state (TS) for an ideal CV.^25^

In this framework, nonphysical jumps in *s*(**z**) can occur at path updates or in regions of the path with high curvature when the system shortcuts the path. While in WTM simulations, this only causes mild heating due to the discontinuity of the bias potential, it can cause numerical instability with the WTM-eABF sampling algorithm due to the coupling to the extended variable. We solve this problem by correcting the position of λ at time step *t* – 1 before integrating its position to time step *t* according to
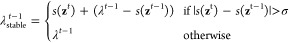
9

**Figure 1:**
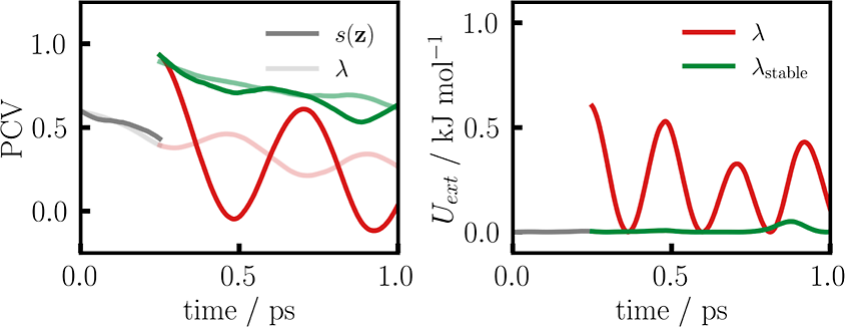
Numerical example for sampling a discontinuous CV with extended-system dynamics. On the left, the trajectories of *s*(**z**) and λ are indicated by solid and transparent lines, respectively. After a step in the PCV, the conventional and stabilized trajectories are given in red and green, respectively. On the right, the corresponding coupling potential *U*_ext_ mediated by harmonic coupling of λ to *s*(**z**) is shown.

We always use the thermal coupling width σ as a threshold for corrections of λ^*t*^, which we find to be a very robust choice. A numerical example of the effect of eq 9 is given in Figure 1. Without the correction, large fluctuations of the extended variable, shown in red on the left, arise after the CV (gray) is stepped. In contrast, by correcting the position of the extended variable, its dynamics (green) are not affected, and the harmonic coupling potential, shown on the right, is continuous, which results in the superior stability of path WTM-eABF simulations.

Additionally, it was shown that a mild confinement of the distance *d*(**z**) from the path, defined by
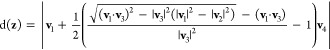
10where **v**_4_ = **z**_*m*_ – **z**_*m*–1_ connects the closest to the second-closest path node, can reduce path shortcutting^28,29^ and speed up the convergence of specific reaction channels.^18^

The central idea of path MtD/WTM is that once the path converges, the bias potential self-corrects as new Gaussians bury artifacts of sampling along the wrong path. Due to the complementary nature of both biasing strategies of the composite WTM-eABF method, simultaneously filling free energy basins and removing barriers,^20^ we expect this self-correction to be much faster. In addition, to further accelerate the convergence of simulations along adaptive paths, we propose a reweighting procedure. For this purpose, the continuously sampled WTM-eABF trajectory is divided into *N* biased states with constant λ = λ_*i*_ and a time-independent potential energy function
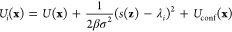
11where *U*_conf_(**x**) denotes some additional confinement potential, as for the distance from the path d(**z**).^30^ This post hoc separation of the biased probability density ρ^*B*^(*z*) into overlapping λ-conditioned distributions ρ^*B*^(*z*|λ_*i*_) allows for the application of popular estimators of the unbiased statistical weights of individual frames like the MBAR.^22^ Note that technically the windows λ_*i*_ must be built separately for each intermediate path if it changes in updates. Besides removing potential artifacts that arise due to the application of confinements on d(**z**), this allows for reweighting of the PMF to any CV of choice. In the context of adaptive PCVs, we suggest applying this formalism to accelerate convergence of the PMF by mapping all data points to the final MFEP. Therefore, assuming that the underlying phase space is already sufficiently sampled, one instantly obtains the correct PMF for a new path without waiting for convergence of the WTM-eABF bias potential. Additionally, other properties like ensemble averages can be recovered independently of the CV, and path updates do not slow down their convergence.

## Computational Details

### Numerical Simulations

As a numerical test, we apply path WTM-eABF to the dynamics of a particle in a 2D MB potential, which is given by
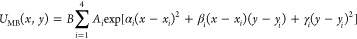
12with *B* = 1 kJ/mol. Other numerical parameters are given in the Supporting Information. For each simulation, a single particle of mass 200 au was evolved in *U*_MB_(*x*, *y*) for 500 ps (1,000,000 steps) according to Langevin dynamics at 50 K with a friction constant of 0.001 fs^–1^. MtD bias potentials were built from Gaussians with a standard deviation of 0.05 and an initial height of 0.01 kJ/mol, which were added every 25 steps. For WTM potentials, the height of new Gaussians is scaled down over the course of the simulation with an effective temperature of 5000 K. For path WTM-eABF, a fictitious particle was coupled to PCVs with σ = 0.01 and mass 25 au. The ABF was scaled with a linear ramp and only fully applied in bins with more than 50 samples. In all simulations, the bias force was accumulated on a grid with a bin width of 0.01. A guess path with 30 nodes was generated by linear interpolation between both minima. For adaptive path simulations, the path was updated every 10 ps according to eq 6. The minimum energy path (MEP) was obtained as a reference by optimizing the guess path with the nudged elastic band (NEB) method.^27^ Scripts to repeat all numerical simulations are given in the Supporting Information.

### Reaction Mechanism of PUS

The initial configuration of the enzyme–substrate complex was taken from the crystal structure of *Pyrococcus furiosus* box H/ACA PUS.^31,32^ Two different crystal structures (PDB codes: 3HJW and 3HAY) were combined to minimize possible errors. The full enzymatic system contains 4 protein subunits (Cbf5, Gar1, Nop10, and L7Ae), as well as a guide and a substrate RNA. The uridine (U) unit in the active site was manually modified from 5-fluorouridine to native U. Charged amino acids were titrated to neutral pH using the H++ program^33^ and placed in a cubic water box containing about 17,000 water molecules. To neutralize the system and set the physiological salt concentration, Mg^2+^ and Cl^–^ ions were added to the water box.

For classical MD simulations, the AMBER-ff19SB force field^34^ was applied together with improved RNA parameters by Tan et al.^35^ and the OPC 4-point water model.^36^ MD simulations were performed with the OpenMM program package.^37^ Electrostatic interactions were calculated by using periodic boundary conditions and particle mesh Ewald summation with a cutoff of 12 Å. Water molecules and H-bonds were constrained using the SETTLE^38^ and SHAKE^39^ algorithms, respectively. Time integration was performed at 300 K with a Langevin integrator using a time step of 2 fs and a friction constant of 1 ps^–1^. Atmospheric pressure was set using a Monte Carlo Barostat.^40^ The initial system was minimized to a tolerance of 10 kJ/mol and carefully heated from 5 to 300 K over 60 stages, 1000 steps each. Afterward, the system was simulated for 600 ns. In the period from 100 to 300 ns, the temperature was increased to 320 K to enhance sampling and enable penetration of water into the active site. Further details are provided in the Supporting Information.

**Figure 2:**
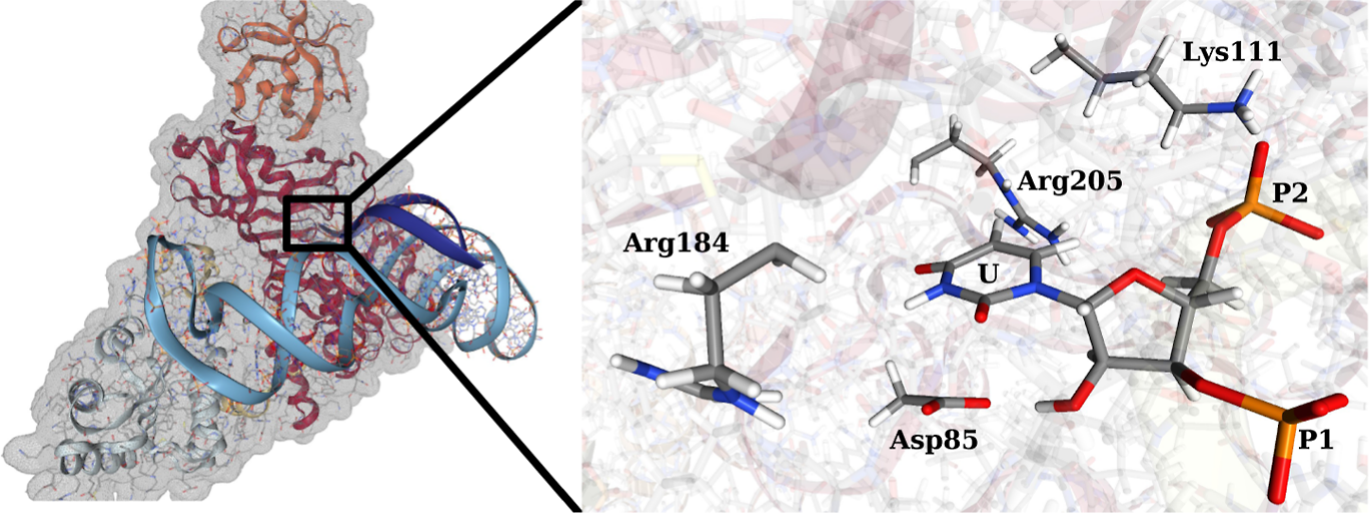
Fully functional *P. furiosus* box H/ACA PUS. The protein–substrate complex contains 4 protein subunits (red: catalytic Cbf5, orange: Gar1, yellow: Nop10, and gray: L7ae), as well as a H/ACA guide and substrate RNA, shown in light and dark blue, respectively (explicit water not shown). On the right, the active site is enlarged. All solid atoms are treated quantum mechanically in QM/MM simulations, while the rest of the protein–substrate complex is included in the MM region. Besides the substrate U, the QM region includes all charged protein residues of the active site, namely, Asp85, Lys111, Arg184, and Arg205.^55^ To balance the charge of the QM region, two phosphates of the RNA backbone are included.

QM/MM simulations were performed in our in-house program package FermiONs++.^41−45^ The QM system was centered inside the simulation box, and interactions between the QM and MM subsystems were treated with electrostatic embedding using a cutoff of 10 Å.^46^ For technical reasons, the TIP3P water model^47^ was applied instead of the OPC. For efficient evaluation of the QM/MM Hamiltonian Grimme’s PBEh-3c DFT functional^48^ was applied. Significant further speed-ups were achieved by fast evaluation of seminumerical exact exchange with the sn-LinK method^45,49^ and evaluation of the Coulomb energy in the RI-J approximation.^50^ Additionally, SCF convergence acceleration was achieved by using accurate guess densities obtained from the previous nine density matrices according to the extended-Lagrangian extrapolation method.^51,52^ The final QM/MM system, which is shown in Figure 2, contained a total of 99 QM and more than 100,000 MM atoms. Geometry optimizations of the QM/MM system were performed in a PyChemshell^53,54^ interface to FermiONs++.

Unconstrained ab initio MD simulations were performed with a time step of 0.5 fs. The temperature was controlled with a Langevin thermostat at 300 K. All atoms farther than 20 Å from the QM region have been frozen. A benchmark of the influence of the PBEh-3c functional, the QM size, and the electrostatic cutoff on the reaction energy of C1′–N1 bond cleavage and proton transfer from H2′ to Asp85-O is given in the Supporting Information.

For the calculation of PMFs, our own Python-based implementation of the path WTM-eABF-enhanced sampling algorithm and MBAR was applied. The full source code is available in the adaptive-sampling package^30^ at https://github.com/ochsenfeld-lab/adaptive_sampling. PMFs were calculated from 10 independent walkers starting from different protein conformations that were picked from the last 100 ns of the MM MD trajectory in an equidistant manner. Trajectories of simulations of the rebound and glycal schemes extend to a combined total of >600 ps and >500 ps, respectively. Reaction and activation-free energies were obtained from the PMF as proposed by Dietschreit and co-workers.^25,56^ More details are given in the Supporting Information.

## Results and Discussion

### Path WTM-eABF on a Numerical Potential

**Figure 3:**
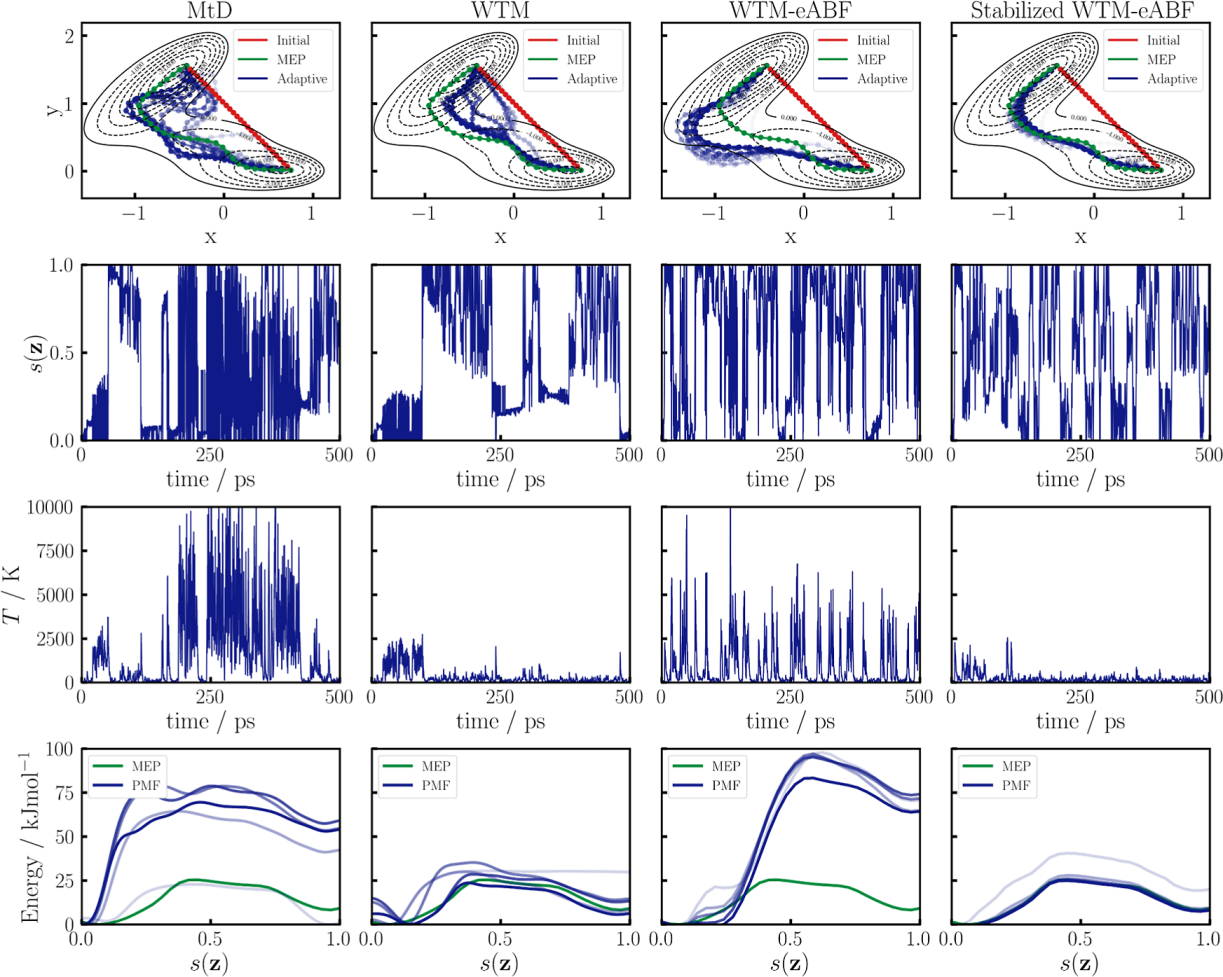
Sampling of a 2D MB potential with adaptive PCV and four different sampling algorithms, MtD and WTM in the first two columns and conventional WTM-eABF and stabilized WTM-eABF in the third and fourth column, respectively. In the top row, the MB potential is shown as a contour plot, with the initial path guess in red and the optimized MEP in green. Snapshots of the adaptive path in 100 ps intervals are shown in blue, losing transparency over time. In the second and third rows, the trajectory of the PCV and rolling temperature average taken over 1 ps are shown, respectively. The last row shows the current PMF estimation every 100 ps, earlier PMFs being more transparent, the potential energy along the MEP shown in green.

We first demonstrate the benefits of the path WTM-eABF algorithm compared to conventional MtD/WTM for a 2D MB potential. We simulate the dynamics of a single particle at 50 K for 500 ps with MtD, WTM, WTM-eABF, and the stabilized variant of WTM-eABF. The MB potential energy surface is shown as a contour plot in the first row of Figure 3. We apply an adaptive PCV as described in Section 2. The initial path, colored red, is a linear interpolation between both minima. We note that for the MB potential the MEP and MFEP are identical, except for small thermal fluctuations of the latter, since the MB energy surface is harmonic in orthogonal direction to the MEP. For the same reason, the analytical probability density is sharply peaked along the MEP and the PMF along the MFEP is approximated well by the potential energy curve along the MEP. Therefore, we use the MEP as a reference, which is shown in green. In blue, snapshots of the adaptive path indicate its evolution over the course of the simulation. The lower rows of Figure 3 show trajectories of the PCV, running temperature averages, and the evolution of the PMF in 100 ps intervals with reference to the MEP potential energy curve (green). The system is initialized in the left minimum at *s*(**z**) ≈ 0. Note that the initial path guess is orthogonal to the MEP. Therefore, in MtD and WTM simulations, a steep bias potential is built initially until this orthogonal barrier can be crossed. The system escapes the first minimum at about 50/100 ps for MtD/WTM, respectively. With MtD, large temperature fluctuations are observed throughout the simulation because of the constant addition of repulsive Gaussian hills that drive the system out of equilibrium. In contrast, the WTM simulation is stable after the first 100 ps, as new Gaussians are scaled down. However, the artificial bias potential that builds up in the initial 100 ps hinders the back reaction to the first minimum, which occurs only shortly before the end of the simulation. Therefore, the adaptive path and also the PMF, which are estimated directly from the bias potential, are both not fully converged after 500 ps. Without stabilization (third column of Figure 3), the WTM-eABF simulation shows large temperature fluctuations as well that cause significant artifacts to the adaptive path and hinder the convergence of the PMF. As discussed in Section 2, these fluctuations are caused by the heating of the extended variable due to the discontinuous nature of the PCV at the path updates. In contrast, with the proposed WTM-eABF stabilization (eq 9), the adaptive path as well as PMF converge rapidly and reproduce the reference almost exactly after less than 200 ps. The dramatically increased performance compared to MtD/WTM can be attributed to the faster adaptation of the WTM-eABF algorithm to path updates due to the combination of two complementary biasing strategies. We note that it is still advisable to perform frequent path updates to avoid the accumulation of large bias potentials along a bad initial guess path. Overall, this shows that the (stabilized) path WTM-eABF is able to significantly outperform MtD/WTM both in terms of robustness and sampling efficiency.

**Figure 4:**
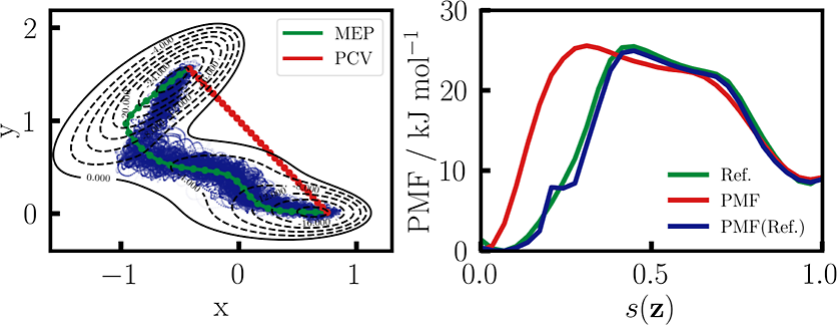
WTM-eABF sampling along a static linear path. On the left, the MB potential energy surface is shown as a contour plot, with PCV nodes in red and the converged MFEP in green. Data points of the WTM-eABF simulation are given in blue. On the right, a reference PMF is shown in green, together with the PMF along the linear guess path in red. The PMF along the MFEP, obtained by reweighting the simulation with MBAR, is given in blue.

In the following, we discuss additional benefits that arise from the combination of WTM-eABF sampling with postprocessing using the MBAR. To this end, we sample along a static, linear PCV using WTM-eABF. On the left of Figure 4, the MB potential is shown along with the PCV and the reference MFEP. Additionally, data points of the trajectory are given in blue. The PMF along the PCV in red on the right of Figure 4, shows large deviations from the reference PMF along the MFEP. However, MBAR allows for the mapping of data points to any reaction coordinate of choice. Therefore, by reweighting the simulation to the MFEP, the reference PMF can be largely recovered. Only at *s*(**z**) ≈ 0.2 an artifact arises, where the sampling has no overlap with the MFEP. In general, this shows that, as the MBAR returns the unbiased weight of each data frame, properties like reaction free energies or ensemble averages are calculated from data frames alone. Therefore, there is no strict need to converge the bias potential after path updates, which, in turn, does not reduce the convergence rate of WTM-eABF/MBAR. In the next section, we will show the beneficial properties of the path WTM-eABF sampling algorithm on a real biocatalytic reaction mechanism.

### Reaction Mechanism of PUS

**Figure 5:**
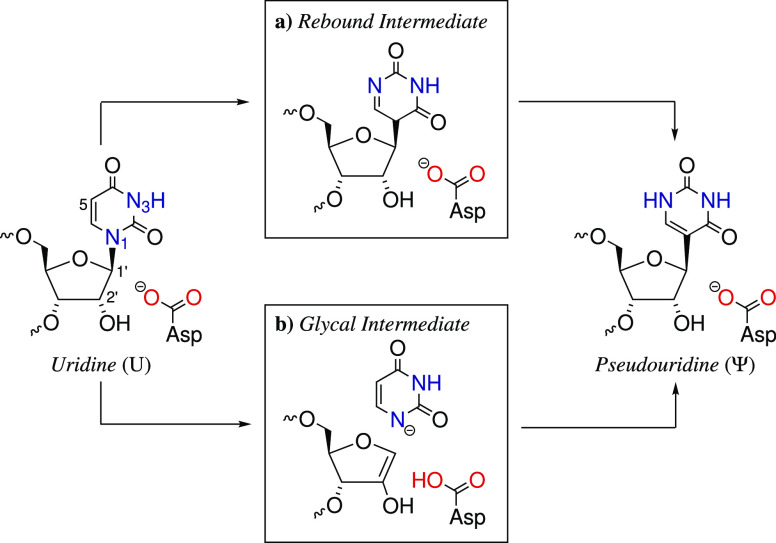
Suggested reaction mechanisms for the conversion of *U* to Ψ catalyzed by PUS. The reaction might run over (a) a rebound intermediate, where the C1′–C5 bond forms directly after C1′–N1 bond cleavage, and (b) a glycal intermediate involving deprotonation of C2′ by Asp85.

To show how the path WTM-eABF method can be applied to explore enzymatic reaction mechanisms, we investigate the first step of the catalytic mechanism of PUS, which enables the site-specific modification of U to pseudouridine (Ψ) in various types of RNA. Because of the highly conserved active site of this family of proteins, which always includes an essential aspartate (Asp85), it is assumed that all PUS enzymes operate by one uniform reaction mechanism.^55^ Recently, in products of PUS, besides the major *ribo* sugar, an *arabino* isomer, which differs in the stereochemistry at C2′, was observed.^57^ Additionally, a large kinetic isotope effect for the proton at C2′ was reported.^58^ Both observations could be explained by the reaction over a glycal intermediate.^58,59^ However, it was also suggested that the reaction instead proceeds over a direct rebound scheme and that the catalytic role of Asp85 is merely to provide conformational strain to the ribo sugar.^60^ An overview of both reaction mechanisms is given in Figure 5.

**Figure 6:**
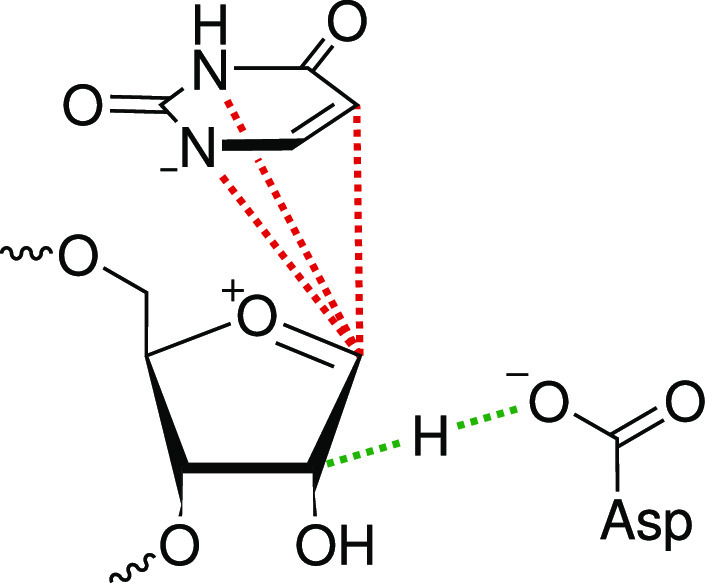
Illustration of the CV space for the calculation of PCVs for the PUS reaction mechanism. For the rebound mechanism, three bond distances marked in red are used. The glycal mechanism additionally involves proton transfer from C2′ to Asp85, with corresponding bond distances marked in green.

We apply path WTM-eABF together with QM/MM MD to explore the formation of both intermediates using the relatively cost-effective PBEh-3c DFT functional and 99 QM atoms. This setup is chosen to reach total simulation times of hundreds of picoseconds, which are necessary to converge PMFs for such a large system. However, we note that due to the inherent inaccuracy of the PBEh-3c functional and the limited QM region size, absolute reaction barriers tend to be overestimated. CV spaces for the calculation of PCVs contain 3 or 5 bond distances, respectively. The breaking U C1′–N1 and forming Ψ C1′–C5 bonds are always included. Additionally, we add the C1′–N3 bond to gain optimal control over the rotation of U. To describe the mechanism over a glycal intermediate, additional slow degrees of freedom that account for proton transfer from C2′ to Asp85 are taken into account. A schematic representation of the CV spaces is given in Figure 6.

#### Rebound Mechanism

**Figure 7:**
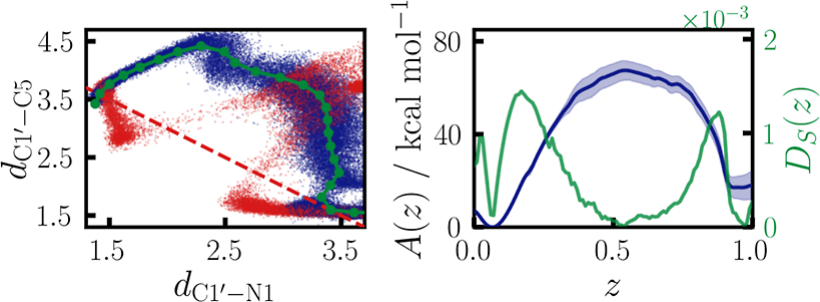
On the left, a 2D projection of path nodes on *d*_C1′–N1_ and *d*_C1′–C5_ is shown in green, with every 20th data point of all combined path WTM-eABF trajectories in blue. Red points denote data points obtained for sampling along a CV of the form ξ(**x**) = *d*_C1′–N1_ – *d*_C1′–C5_ (indicated by the dashed red line). On the right, the PMF obtained from all combined simulations is shown in blue; in green, the orthogonality measure *D*_*S*_(*z*) (eq 8). Light blue area denotes the 95% confidence interval.

First, we consider the rebound mechanism, as proposed by Kiss et al.,^60^ where C1′–N1 bond cleavage is followed by direct formation of the characteristic Ψ C1′–C5 bond. In Figure 7, on the left, the final path is projected on the C1′–N1 and C1′–C5 bond distances, with trajectory data points shown in blue. On the right are the obtained PMF together with the CV criterion *D*_*S*_(*z*) (eq 8). The obtained reaction mechanism can be divided into three steps. First, both the C1′–N1 and C1′–C5 bonds elongate as U unbinds from the glycase backbone building an uridilate ion. For this step, we obtain a high activation-free energy of about 69 kcal/mol, which can be rationalized by the involved charge separation. At a C1′–N1 bond distance of about 2.5 Å, the uridilate ion begins to rotate under shortening of the C1′–C5 bond distance. Finally, the C1′–C5 bond forms, building the stable rebound intermediate. The CV criterion has three clear minima at the reactant, product, and TS, respectively. That it is zero at the TS indicates the good quality of the obtained PCV and confirms that the TS is successfully located. We note that this rotating motion cannot be fully captured by a simple linear combination of the form *d*_C1′–N1_ – *d*_C1′–C5_, which we mark in red in Figure 7. On the contrary, the application of this very common choice of CV leads to sampling defects. Due to the wrong projection of the bias force, simultaneous *d*_C1′–N1_ elongation and *d*_C1′–C5_ shortening are enforced, which leads to a bending motion until rapid breaking of the C1′–N1 bond occurs. Also, various irrelevant side reactions are observed, like the formation of C1′–O2 bonds, which make simulations unstable. With a mild confinement on the distance from the path such side reactions are fully suppressed in PCV simulations.

#### Glycal Mechanism

**Figure 8:**
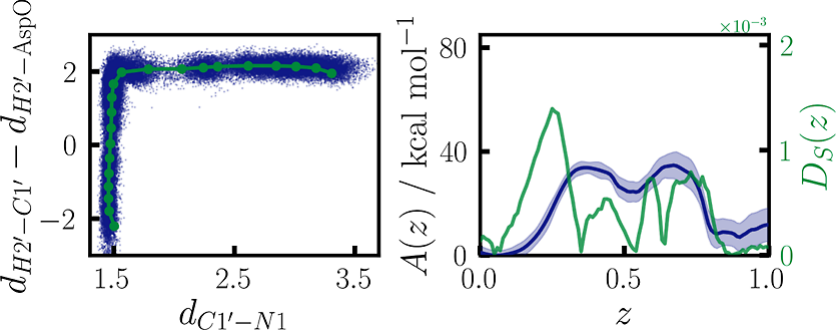
On the left, a 2D projection of path nodes on *d*_C1′–N1_ and *d*_H2′–C1′_ – *d*_H2′–AspO_ is shown in green, with every 20th data point of all combined trajectories in blue. On the right, the obtained PMF is shown in blue, and in green, the orthogonality measure *D*_*S*_(*z*) (eq 8). Light blue area denotes the 95% confidence interval.

The large activation-free energy obtained for the formation of the rebound intermediate indicates that in this mechanism, the catalytic role of Asp85 is not correctly captured. Recently, a large kinetic isotope effect was shown for exchanging H2′ with deuterium, an observation that might be explained by H2′ proton transfer to Asp85, forming a glycal intermediate. Therefore, we build a new path guess, where parts of the final path of the rebound mechanism (without C1′–C5 bond formation) are coupled to proton transfer from C2′ to Asp85. A 2D projection of the new CV space is shown in Figure 8 on the left, with every 20th data point of the simulation shown in blue and the final path in green. Clearly, a sequential mechanism is observed, where proton transfer from C2′ to Asp85 (up direction) is followed by C1′–N1 bond cleavage (left to right). The new PMF and corresponding CV criterion are given in Figure 8, on the right. Two distinct TSs and one intermediate minimum, resembling the deprotonated ribo sugar before C1′–N1 bond cleavage, are observed. Each of them is confirmed by the clear minima of *D*_*S*_(*z*). With an activation-free energy of about 35 kcal/mol, the initial proton transfer is the rate-limiting step and significantly activates sequential C1′–N1 bond cleavage, for which a small remaining activation-free energy of around 10 kcal/mol is obtained. The significant relative reduction of the activation-free energy compared to the rebound mechanism by over 30 kcal/mol displays the large catalytic effect on C1′–N1 bond breaking. At the same time, we concede that the absolute activation barrier obtained is still too high to be crossed on relevant time scales under biological conditions. However, we also expect it to be overestimated due to the inherent inaccuracy of the PBEh-3c functional and the limited size of the QM subsystem (see Supporting Information).

Overall, this shows how adaptive PCVs enable the detailed study of nonlinear, multistep molecular transitions. The above example depicts how their application, together with the highly efficient path WTM-eABF algorithm, facilitates the systematic exploration of reaction mechanisms, even using costly QM/MM simulations. In addition, we show how the adaptive path can directly yield mechanistic information, like the activation of C1′–N1 bond breaking by deprotonation of C2′ in PUS enzymes.

## Conclusions

We have shown the benefits of combining the highly efficient WTM-eABF sampling algorithm with adaptive PCVs. To this end, we provide an implementation of path WTM-eABF with a new stabilization algorithm that ensures the temperature stability of simulations even if path shortcutting or path updates cause sudden jumps in the PCV. Additionally, we show how reweighting data to an updated path with the MBAR can be used to speed up the convergence of PMFs along adaptive paths. Overall, we argue that WTM-eABF, PCVs, and the MBAR estimator elegantly complement each other and together offer a highly competitive approach to the systematic investigation of reaction mechanisms in complicated biochemical systems.

We apply these methods to investigate the reaction mechanism of PUS and show how path WTM-eABF enables the exploration of challenging nonlinear molecular motions like uridine rotation in the rebound mechanism. Furthermore, we obtain a significantly reduced activation free energy for a glycal mechanism where proton transfer of H2′ to the essential Asp85 activates C1′–N1 bond breaking, a result that is in line with the experimental observation of a large deuterium kinetic isotope effect for H2′.^58^ In a future study, we plan to use the presented framework to provide a full mechanistic picture of PUS.

## Supporting Information Available

The data that supports the findings of this study are available from the corresponding author upon reasonable request. The Supporting Information is available free of charge at https://pubs.acs.org/doi/10.1021/acs.jctc.3c00938.

Additional information on the implementation of PCVs, numerical parameters of the MB potential, initial MM simulation of PUS, accuracy benchmarks for the QM/MM setup, and details on QM/MM free energy simulations (PDF)
Python scripts to repeat and analyze MtD, WTM, and WTM-eABF simulations along static and adaptive PCVs on a numerical MB potential (ZIP)
