# Supplementary material for: Improved Sampling of Adaptive Path Collective Variables by Stabilized Extended-System Dynamics
Source: J Chem Theory Comput. 2023 Dec 11;19(24):9202–10. doi: 10.1021/acs.jctc.3c00938 (PMC10753802; doi:10.1021/acs.jctc.3c00938)
Supplement: Supplementary file 1 — ct3c00938_si_001.pdf [file ct3c00938_si_001.pdf]

Supporting Information: Improved Sampling of Adaptive Path Collective Variables by Stabilized Extended-System Dynamics

Andreas Hulm,[1] Christian Ochsenfeld[1,2,*]

[1]Chair of Theoretical Chemistry, Department of Chemistry,
University of Munich (LMU), Butenandtstr. 7, D-81377 München, Germany

[2]Max Planck Institute for Solid State Research, Heisenbergstr. 1, D-70569 Stuttgart, Germany

*E-Mail: christian.ochsenfeld@uni-muenchen.de

Contents

1 Implementation of Path Collective Variables S2

2 Numerical Parameters for the Müller-Brown Potential S3

3 Initial MM Simulation of the Enzyme-Substrate Complex S3

4 QM/MM Accuracy S4

5 Details on Calculations of PUS Reaction Mechanisms S6

1 Implementation of Path Collective Variables

In practice, to avoid numerical problems if the system leaves the path ($s(\mathbf{z}) < 0$ or $s(\mathbf{z}) > 1$) we add one boundary node at each side by linear extrapolation of the outer two nodes. Therefore, eq. 5 of the main manuscript is calculated including the boundary nodes and m/M is replaced by $(m-1)/M$ to ensure that $s(\mathbf{z}) = 0$ if $\mathbf{z}$ is equal to the first original node vector and $s(\mathbf{z}) = 1$ if $\mathbf{z}$ is equal to the last original node vector. Additionally, to keep the system in the range of interest in path WTM-eABF simulations, the fictitious particle λ is always confined to the range $0 <= s(\mathbf{z}) <= 1$ with harmonic wall potentials.

One inherent problem of PCVs is there dependence on the definition of path nodes. If the nodes along the path are ill-defined, for example due to large kinks in the path or cluttering of parts of the path with multiple nodes, the PCV will be ill-defined as well. Therefore, it is necessary to perform a reparametrization step after every path update [1]. First, the path is smoothed according to

$$\mathbf{z}_i^* = (1-s)\mathbf{z}_i + \frac{s}{2}(\mathbf{z}_{i-1} + \mathbf{z}_{i+1}) \tag{S1}$$

where s denotes a damping factor for the smoothing which ranges from 0 (no smoothing) to 1 (linear interpolation between neighbor nodes). Second, equidistant spacing of nodes is ensured via

$$\mathbf{z}_i^{**} = \mathbf{z}_{j-1}^* + (s(i) - L(j-1))\frac{\mathbf{z}_j^* - \mathbf{z}_{j-1}^*}{|\mathbf{z}_j^* - \mathbf{z}_{j-1}^*|}. \tag{S2}$$

Here $s(m) = (m-1)\frac{L(N)}{N-1}$, with total length of path $L(N)$, denotes the position of node m on the path under equidistant spacing and $L(m)$ is actual position of node m along the path. Index k is such that $L(k-1) < s(m) \leq L(k)$. Eq. S2 is iterated until the change in the total path length drops below some tolerance (e.g. 0.001).

2 Numerical Parameters for the Müller-Brown Potential

The 2D Müller-Brown potential energy surface is defined by

$$U_{MB}(x, y) = B \sum_{i=1}^{4} A_i \exp\left[\alpha_i(x - x_i)^2 + \beta_i(x - x_i)(y - y_i) + \gamma_i(y - y_i)^2\right] \tag{S3}$$

with $B = 1\,\text{kJ/mol}$ and other numerical parameters given in Table S1.

Table S1: Applied parameters of MB potential.

i	A_i	α_i	β_i	γ_i	x_i	y_i
1	-40.0	-1.0	0.0	-10.0	1.0	0.0
2	-10.0	-1.0	0.0	-10.0	0.0	0.5
3	-34.0	-6.5	11.0	-6.5	-0.5	1.5
4	3.0	0.7	0.6	0.7	-1.0	1.0

3 Initial MM Simulation of the Enzyme-Substrate Complex

It has been shown experimentally that deprotonation or reprotonation of C2' is partially rate limiting for the reaction mechanisms of PUS. Furthermore, experimental evidence points towards direct deprotonation by the nearby catalytic Asp85 [2]. Therefore, we consider the AspO-C2'H distance as a first indicator of potential reactivity. Specifically, only configurations where this distance is smaller than 4 Å are considered as suitable starting points for further QM/MM investigations of the reaction mechanism.

The top left panel of figure S1 shows the rolling average of the temperature over the course of a 600 ns MM-MD simulation. Between 100 and 300 ns the temperature is increased to enhance sampling and enable penetration of the water into the active region. The backbone RMSDs of individual protein subunits shown in the bottom left panel are not affected by the heating period and always below 2 Å. Finally, the top right panel shows the AspO-C2'H distance. During the first 100 ns the system is trapped in a metastable unreactive state because of electrostatic attraction of the negatively charged Asp85 to the nearby positively charged Arg184. After 100 ns upon heating of the system to 320 K the mobility of Asp85 increases, and it enters a second metastable configuration. The electrostatic attraction of Asp85 to Arg184 is reduced by water that has penetrated the active site in the first few hundred ns of the simulation, while a stabilizing H-bond to O2'H of the nucleoside backbone forms. Thus, 10 equidistant snapshots are taken from the last 100 ns of the MM MD simulation for further QM/MM simulations. Overall, the final structure agrees well with the X-Ray structure of the active site, which confirms that these configurations can be regarded as realistic starting point for the mechanistic study.

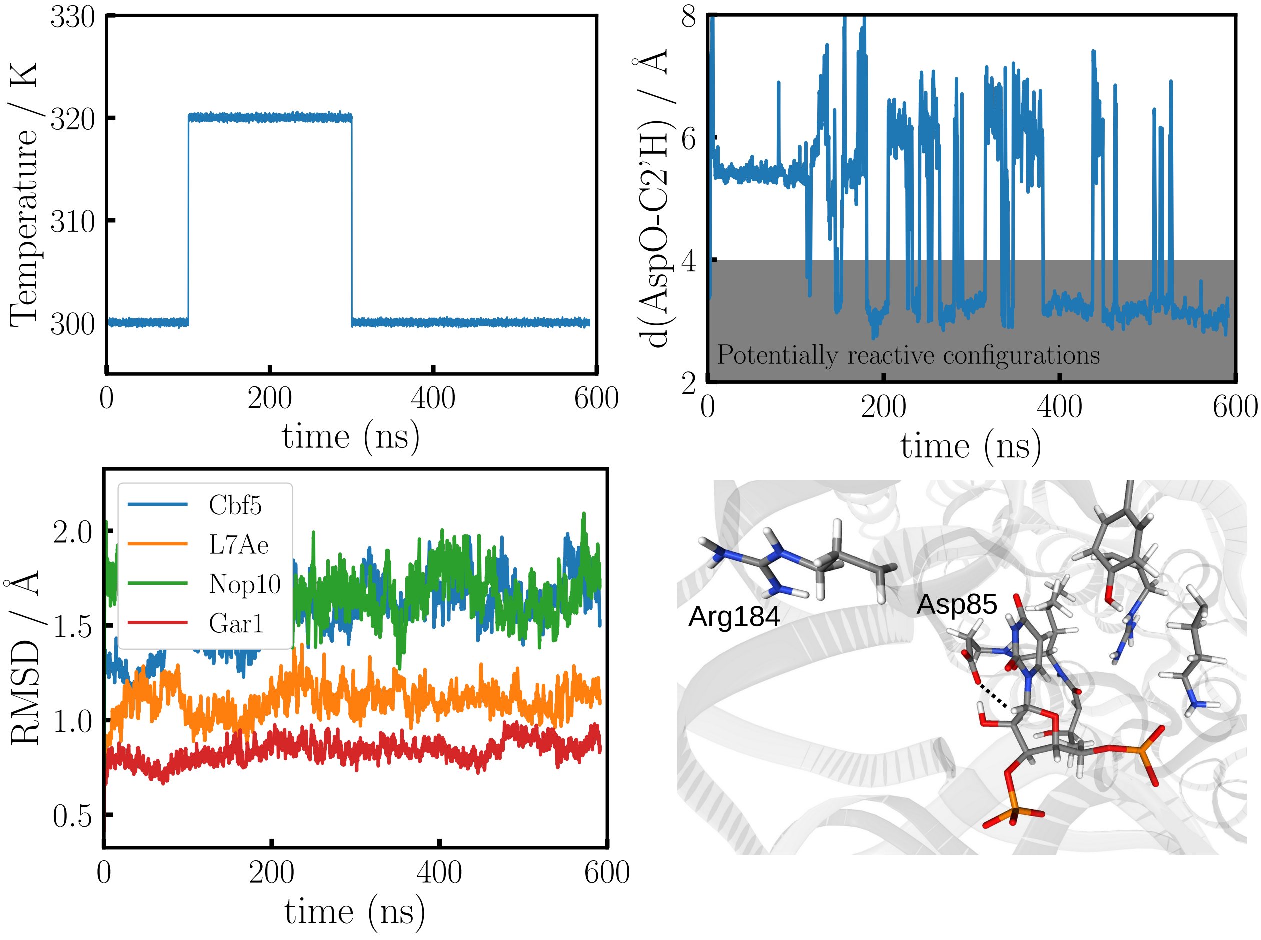

Figure S1: On the top left the rolling average of the temperature during 600 ns MM-MD simulation is shown. In the time period from 100 to 300 ns the system is heated from 300 to 320 K to enhance sampling. The bottom left panel shows the rolling average of the individual backbone RMSDs of all 4 protein subunits. Cbf5 in blue, L7Ae in orange, Nop10 in green and Gar1 in red. On the top right the rolling average of the O-H distance between Asp85-O1/O2 and U-C2'H during 600 ns MM-MD simulation. To take into account the rotation of the carboxyl group always the distance to the closer carboxylic oxygen is plotted. The active site of the protein is shown on the bottom right, with the target U, the negatively charged catalytic Asp85 and the nearby positively charged Arg184. The dashed line shows the AspO-C2'H distance.

4 QM/MM Accuracy

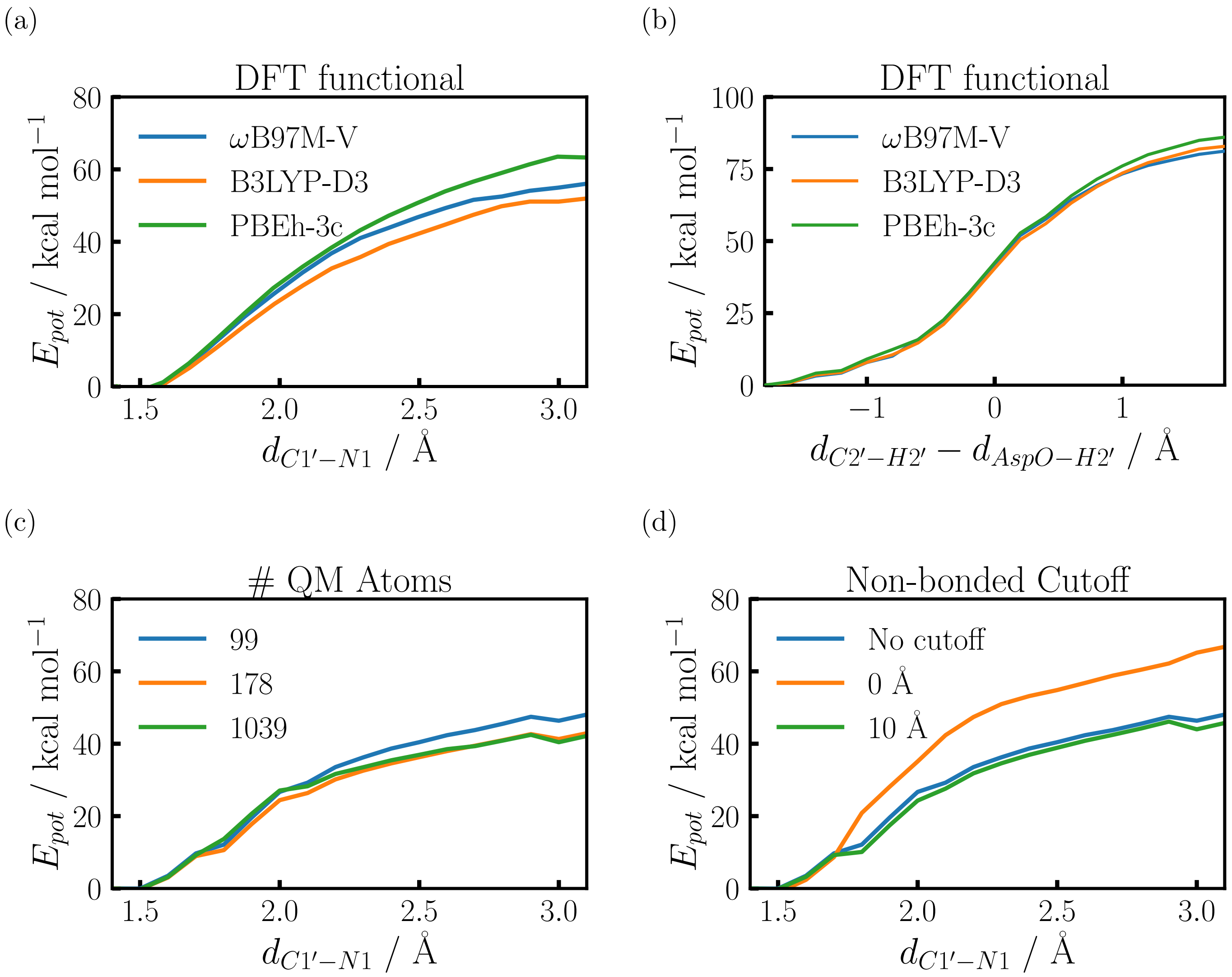

Figure S 2: Benchmark calculations on minimum energy paths (MEPs) of the C1'-N1 bond cleavage and proton transfer reaction of H2' to AspO. (a,b) For different DFT functionals on a single ribo-nucleotide implicitly solvated in water (COSMO [3]). For (b) also all charges residues in 5 Å proximity and two phosphates of the RNA backbone are added. Shown are MEPs computed using the highly accurate ωB97M-V functional [4, 5], the popular B3LYP-D3 functional [6, 7] (both with the triple-ζ basis set def2-TZVP [8]), and Grimme's cost-effective PBEh-3c functional [9]. Due to its much higher cost efficiency we will use PBEh-3c for *ab-initio* molecular dynamics simulations, which tends to overestimate both reaction barriers. By this choice resulting reaction (free) energies might safely be regarded as upper bound to the true energy. (c) For the number of QM atoms used in QM/MM simulations. The smallest region with 99 atoms contains the ribo-nucleotide in the active site together with all charged residues in 5 Å proximity, while the largest QM region with 1039 contains all atoms within 5 Å. The medium sized QM region with 178 atoms is designed to contain all important interactions in the active site and reproduce the energy of the largest QM region. No cutoff is used for electrostatic interactions with the MM system. (d) For the influence of the cutoff of electrostatic interactions with the MM subsystem. With a cutoff of 0 Å the electrostatic interaction of the QM region with the environment is switched off, while using no cutoff corresponds to inclusion of electrostatic interaction with all MM atoms. Using a cutoff of 10 Å preserves high accuracy while offering about 4-fold speedup of the calculation with reference to no cutoff. All QM/MM calculations are performed on one snapshot of the full system taken from the last 100 ns of an MM-MD using the PBEh-3c functional. For (d) the medium-sized QM region with 178 atoms is applied.

5 Details on Calculations of PUS Reaction Mechanisms

Reaction free energy profiles are calculated using QM/MM-MD simulations. A time step of 0.5 fs is used, and the temperature is controlled at 300 K using Langevin dynamics with a friction constant of 0.001 fs^{-1}. The exploration of reaction coordinates is accelerated with the Well-Tempered Metadynamics extended-system Adaptive Biasing Force (WTM-eABF) hybrid algorithm [10]. For the WTM potential, Gaussian's with an initial height of 1 kJ/mol and variance of 0.03 were deposited every 10 fs. The effective temperature was set to 4000 K. The fictitious particle had a mass of 40 a.u. and was coupled to the CV with a thermal coupling width of 0.01. The WTM and ABF forces were collected on a grid with bin width 0.01. The ABF force was scaled up with a linear ramp and fully applied in bins with more than 200 samples. Our own implementation of adaptive PCVs [11, 12] is used to describe transitions associated with different reaction mechanisms. The distance from the path is confined with a harmonic potential with a force constant of 100 kJ/molÅ^2 to suppress potential side reactions. For the glycal mechanisms still side reactions are observed due to the high mobility of H2', which are filtered out in post-processing. Observed side reactions include protonation of the uridine N1 with H2' and exchange of the hydroxy O2'H (which is hydrogen bonded to the catalytic Asp85) with H2'. Additionally, frames with a confinement force to the path larger than 60 kcal/mol are removed to only consider conformations that are reasonably close to the path. This leads to a slightly reduced total initial simulation time of around 530 ps compared to the rebound mechanism with over 600 ps.

Table S 2: Bond distance thresholds for filtering of frames in post-processing of simulations of the glycal mechanism.

	Threshold
$d_{H2'-N1}$	< 2.00
$d_{O2'-O2H'}$	> 1.25

To get optimal control over path convergence 10 walkers are simulated for 5-20 ps each at a time and the update is calculated manually from the full data. PCVs are defined in the space of a small set of bond distances, that are suitable to describe the slow degrees of freedom of the given process (see Table S3). Here, the distances of H2' to Asp85 oxygens is defined as

$$d_{\mathrm{AspO-H2'}} = \min\left[d_{\mathrm{Asp_{85}O1-H2'}}, d_{\mathrm{Asp_{85}O2-H2'}}\right] \tag{S4}$$

Note, that $d_{\mathrm{AspO-H2'}}$ is smooth even if Asp85 rotates during the simulation and the proton acceptor oxygen changes.

Table S3: Bond distances that build the CV space for calculations of PCVs.

	Rebound	Glycal
C1'-N1	✓	✓
C1'-C5	✓	✓
C1'-N3	✓	✓
C2'-H2'	×	✓
AspO-H2'	×	✓

Final free energy profiles are obtained from WTM-eABF biased trajectories using the MBAR estimator [13], as proposed in Ref. [14]. The MBAR equations are solved self-consistently. The starting guess for reduced free energies $\beta\hat{f}_i$ is zero and $\hat{f}_1$ is set to zero after every cycle. Convergence is reached when the largest change of $\beta\hat{f}_i$ compared to the last cycle drops under 10^{-6}. Reaction and

activation free energies are estimated from the PMF as proposed in Refs. [15, 16]. Confidence intervals are calculated from the standard deviation between independent simulations. The full source code is available on Github under `https://github.com/ochsenfeld-lab/adaptive_sampling`.

References

[1] Maragliano, L., Fischer, A., Vanden-Eijnden, E., Ciccotti, G., *J. Chem. Phys.* **2006**, *125*, 024106.

[2] Veerareddygari, G. R., Singh, S. K., Mueller, E. G., *J. Am. Chem. Soc.* **2016**, *138*, 7852–7855.

[3] Klamt, A., *Wiley Interdiscip. Rev.-Comput. Mol. Sci.* **2011**, *1*, 699–709.

[4] Mardirossian, N., Head-Gordon, M., *J. Chem. Phys.* **2016**, *144*, 214110.

[5] Mardirossian, N., Head-Gordon, M., *Mol. Phys.* **2017**, *115*, 2315–2372.

[6] Stephens, P. J., Devlin, F. J., Chabalowski, C. F., Frisch, M. J., *J. Phys. Chem.* **1994**, *98*, 11623–11627.

[7] Grimme, S., Antony, J., Ehrlich, S., Krieg, H., *J. Chem. Phys.* **2010**, *132*, 154104.

[8] Schäfer, A., Horn, H., Ahlrichs, R., *J. Chem. Phys.* **1992**, *97*, 2571–2577.

[9] Grimme, S., Brandenburg, J. G., Bannwarth, C., Hansen, A., *J. Chem. Phys.* **2015**, *143*, 054107.

[10] Fu, H., Shao, X., Cai, W., Chipot, C., *Acc. Chem. Res.* **2019**, *52*, 3254–3264.

[11] Leines, G. D., Ensing, B., *Phys. Ref. Lett.* **2012**, *109*, 020601.

[12] Pérez de Alba Ortız, A., Tiwari, A., Puthenkalathil, R., Ensing, B., *J. Chem. Phys.* **2018**, *149*, 072320.

[13] Shirts, M. R., Chodera, J. D., *J. Chem. Phys.* **2008**, *129*, 124105.

[14] Hulm, A., Dietschreit, J. C., Ochsenfeld, C., *J. Chem. Phys.* **2022**, *157*, 024110.

[15] Dietschreit, J. C., Diestler, D. J., Ochsenfeld, C., *J. Chem. Phys.* **2022**, *156*, 114105.

[16] Dietschreit, J. C., Diestler, D. J., Hulm, A., Ochsenfeld, C., Gómez-Bombarelli, R., *J. Chem. Phys.* **2022**, *157*, 084113.
